# EPPOCRATIS: A Point-of-Care Utilization of Virtual Surgical Planning and Three-Dimensional Printing for the Management of Acute Craniomaxillofacial Trauma

**DOI:** 10.3390/jcm10235640

**Published:** 2021-11-29

**Authors:** Basel A. Sharaf, Jonathan M. Morris, Doga Kuruoglu

**Affiliations:** 1 Division of Plastic Surgery, Department of Surgery, Mayo Clinic, Rochester, MN 55902, USA; kuruoglu.doga@mayo.edu; 2 Anatomic Modeling Lab, Department of Radiology, Mayo Clinic, Rochester, MN 55902, USA; morris.jonathan@mayo.edu; 3 Department of Radiology, Mayo Clinic, Rochester, MN 55902, USA

**Keywords:** point of care, virtual planning, three-dimensional printing, facial trauma, maxillofacial fractures

## Abstract

While virtual surgical planning (VSP) and three-dimensional planning (3DP) have become important tools in acute craniomaxillofacial surgery, the incorporation of point of care VSP and 3DP is crucial to allow for acute facial trauma care. In this article, we review our approach to acute craniomaxillofacial trauma management, EPPOCRATIS, and discuss current challenges and future directions in acute facial trauma management.

## 1. Introduction

Virtual surgical planning (VSP) and three-dimensional printing (3DP) have become widely used in planning and execution of craniomaxillofacial surgery [1,2]. Previous studies reported successful use of patient-specific 3DP models and surgical guides in the operating room [2,3,4,5]. However, the process of VSP and 3DP continues to be time-consuming due to the sourcing to third-party companies and lack of in-house computer-aided design and manufacturing (CAD/CAM) processes [2,6]. The integration of VSP and 3DP at the point-of-care (POC) is paramount for a timely and efficient use of the technology in the management of acute craniomaxillofacial trauma.

We previously introduced our approach to complex craniofacial trauma reconstruction; EPPOCRATIS. EPPOCRATIS stands for Expedited Preoperative Point Of Care Reduction of Fractures to normalized Anatomy and Three-dimensional Printing to Improve Surgical Outcomes [6]. In this article, we review the workflow of this innovative approach in detail, focusing on each stage of the EPPOCRATIS process.

## 2. EPPOCRATIS Process

EPPOCRATIS consists of a total of seven stages which are as follows: (1) computed-tomography (CT) scan/data acquisition, (2) segmentation, (3) virtual surgical planning (VSP), 94) 3-Dimensional Printing (3DP), (5) Post-processing, (6) Plate Bending and (7) surgery (Video S1, Supplementary Materials). The entire process can be completed in 24-48 h from CT Scan/Data acquisition to surgery. Most of this time in 3DP model creation involves the printing and post-processing stages.

### 2.1. CT Scan/Data Acquisition

CT images of the facial bones and head are obtained using the standard thin slice, 0.5–0.75 mm, trauma protocol thickness. DICOM images are then transferred to the anatomic modeling unit, which is an in-house point of care manufacturing facility.

### 2.2. Segmentation

The CT images are imported into an advanced image processing software, Mimics 23.0 (Materialise, Leuven, Belgium). The mandible and skull are segmented, a process in which borders of anatomic regions within the scan are defined from 2D greyscale images to create a 3D model. Each individual fracture fragment that will be moved to a normalized position must be segmented as a unique object. Once the segmentation is complete, the segmented file is transferred to a medical CAD software program 3-Matic 15.0 (Materialise, Leuven, Belgium) for 3D file error fixing, optimization, and 3DP file export (Video S1, Supplementary Materials). This stage takes 30–35 min including the time it takes for a radiologist to perform quality control and look at the contours of the 3D file back on the axial images to assure the accuracy of segmentation.

### 2.3. VSP

Once the 3D file is created, a VSP session is performed by the biomedical engineers and the plastic surgeon. In this stage, the virtual reduction of fractures to “normalized anatomy” is ensured by moving the fragments back to their original position using mirror imaging if the contralateral side is normal, using standard anatomic landmarks, or 3D files from age-matched normal compares. The software’s tools such as “transparency” and “mirroring” are utilized to achieve virtual anatomic reduction of the fractures in three planes. The “overlap” tool enables the user to evaluate the reduction clearly by overlapping the post-traumatic and post-VSP 3D-CT images (Video S1, Supplementary Materials). For the occlusal fractures, the dental arch alignment is designed to achieve the optimal anatomical alignment, although minor discrepancies with the true occlusion may still occur and should be corrected intraoperatively. The engineer will spend an average of 20 min on CAD file fixing, mirror imaging, and preparing for printing.

### 2.4. 3D Printing

**Figure 1:**
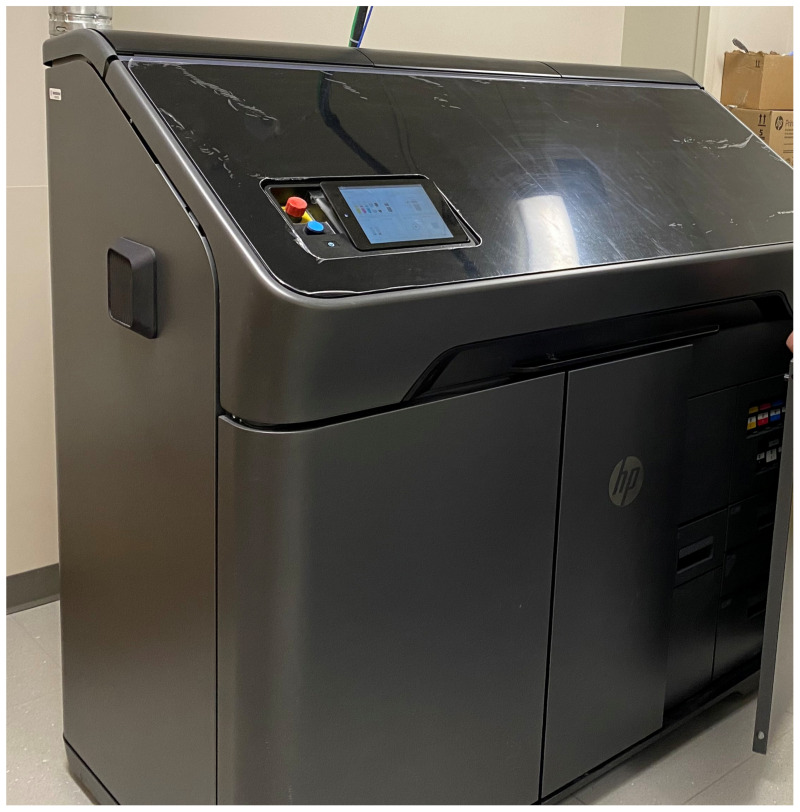
Hewlett-Packard (HP, Palo Alto, CA, USA) 3D Printer.

Following the virtual reduction of the fractures to the normalized anatomy, the 3D image is exported as a .STL (Standard Tessellation Language) file. This file contains the 3D data for “the patient-specific virtually reduced craniofacial model”. The virtual model is then put through slicing software so the 3D object can be manufactured 1 slice at a time by the 3D printer. For full-color non-sterilizable printing that can be used for preoperative planning and education, three printer technologies are available, including binder jetting, material jetting, and sheet lamination. If sterilizable biocompatible 3D printed models are needed for plate bending and intraoperative use, then powder bed fusion and vat polymerization can be used. In our facility, the sterilizable 3DP model is made using vat photopolymerization on a Form3B Form Labs stereolithography (SLA) 3D printer (Formlabs, Boston, MA, USA) using Formlabs Surgical Resin. The 3DP objects made with this resin material have been independently verified by our institution to be sterilizable in the autoclave, biocompatible, and not cytotoxic. The printing stage varies from 2 h to 13 h based on technology and resolution of printer and print technology. (Figure 1).

### 2.5. Post-Processing

After printing, the 3DP model undergoes a post-processing process by the AMU staff prior to being delivered to the surgeon. Depending on the technology used for printing the post-processing step to remove support material can include removing excess powder, bead blasting, soaking in lye baths and water jetting, soaking the model in color binding agents and cyanoacrylate, or photocuring and removal of support material. For Formlabs Form 3B SLA printers using surgical resin, the steps include alcohol washing, photocuring, and finally, support strut removal. Additionally, a quality control step is added to assure anatomic accuracy of the 3D printed model to the 3D printed CAD file. This included visual inspection, surface metrology, and caliper measurements. The post-processing stage takes 15 min to 6 h, depending on the model and material printed.

### 2.6. Plate Bending

**Figure 2:**
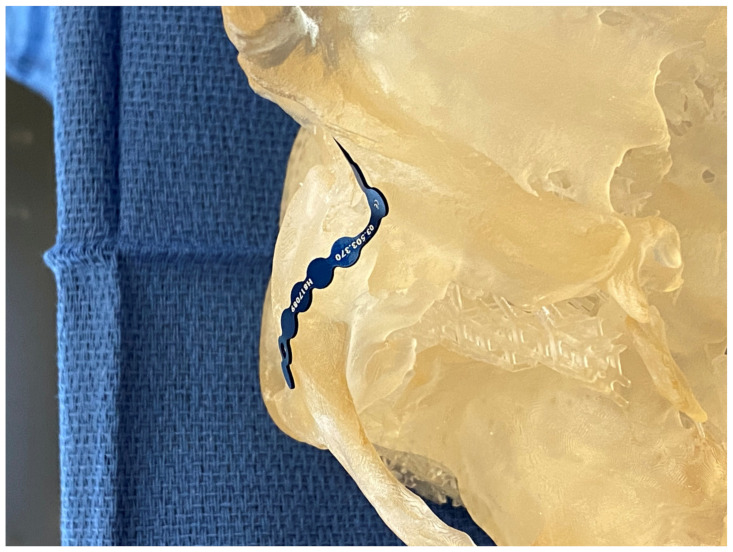
Template bending in preparation for plate bending on a patient-specific high-fidelity 3DP model.

**Figure 3:**
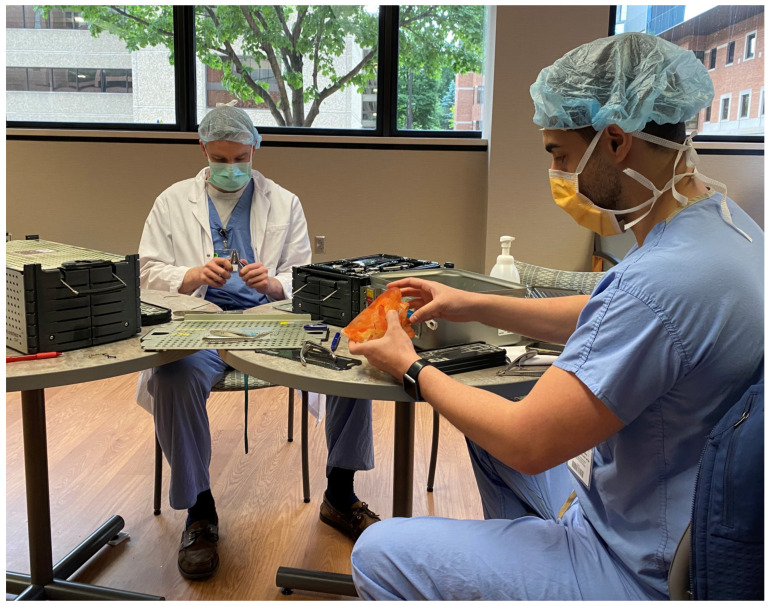
Using the high fidelity 3DP model preoperatively to teach trainees how to bend plates and review pertinent anatomy and surgical plan.

The virtually reduced 3DP model is then delivered to the plastic surgeon for bending the fixation plates in accordance with the 3DP model’s normalized form and shape (Figure 2). This serves as a valuable hands-on training opportunity for the residents outside the operating room pressure (Figure 3) and reduces costly intraoperative time. The prebent plates are then sterilized for intraoperative use. The plate bending time is dependent on the complexity of the case, taking approximately 1–2 h.

### 2.7. Surgery

**Figure 4:**
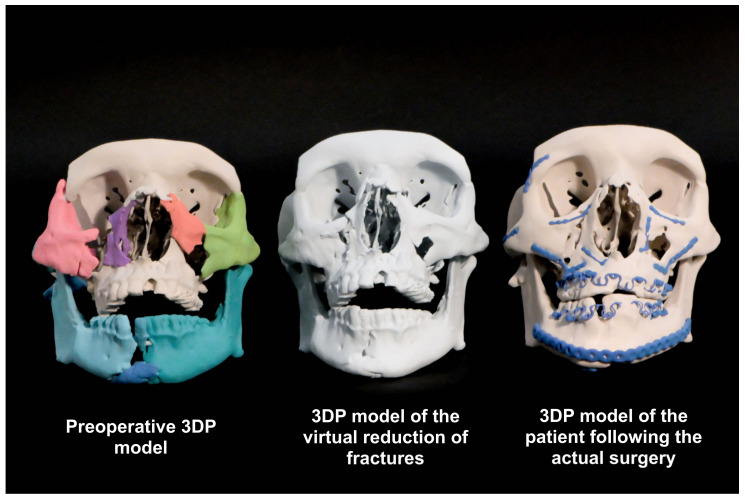
Patient-specific high-fidelity 3DP models from various stages of EPPOCRATIS.

The sterilized prebent plates are used in the operating room as guides. Instead of fracture reduction first, then plate adaptation, the plate serves as a surgical guide for fracture reduction based on the perfected anatomy in the 3DP model. This adds another layer of accuracy to fracture reduction, especially in extensive panfacial trauma. Furthermore, the intraoperative time is shortened with EPPOCRATIS. Perioperative 3DP models of a patient during the EPPOCRATIS process are shown in Figure 4.

## 3. Discussion

Over the past decade, the use of CAD/CAM has become increasingly more utilized in craniomaxillofacial surgical planning and facial trauma management [2,4,6,7,8,9,10,11,12,13].

While surgical accuracy and efficiency have been proposed to be enhanced via these advanced 3D technologies [2,10], there still exist certain challenges with them, including high cost, the time needed for communication with the bioengineers, which may require several virtual meetings, and the lag time from CT scan acquisition to 3DP surgical model production. With the current lag time averaging at least 1–2 weeks for the full process, the implementation of VSP and 3DP in acute facial trauma management has been limited due to outsourcing to third-party companies [6]. Our current experience with reputable third-party vendors when VSP and 3DP is utilized in craniomaxillofacial procedures that require patient-specific guides and implants does not allow the expedited EPPOCRATIS process timeline for acute maxillofacial trauma. The process with third-party vendors takes 1–2 weeks from the initial CT scan in the emergency room to the delivery of final 3D printed model and surgical guides. This lag time is still necessary to transfer the CT imaging data to third-party vendors, segmentation by their bioengineers, scheduling a web meeting for VSP, and review of the plan prior to approval of the final plan and 3D printing. Nevertheless, these challenges can be overcome by utilizing these technologies at the point-of-care; where surgical care of the facial trauma patient is being provided. In this article, we present the first author’s approach to acute facial trauma management at a tertiary academic center using EPPOCRATIS; Expedited Preoperative Point Of Care Reduction of Fractures to normalized Anatomy and Three-dimensional Printing to Improve Surgical Outcomes.

EPPOCRATIS requires a multidisciplinary team consisting of the plastic surgeon, radiologist, bioengineer, and CT technologist. Advanced software for segmentation, VSP, and 3D printers are also required. In a recent study by Bergeron et al. [2], an in-house application of VSP and 3DP was described for acute craniomaxillofacial trauma. The authors reviewed their in-house VSP and 3DP experience in the management of 9 patients with acute facial trauma. They reported a mean printing duration of 7 h and 55 min and a mean filament cost of $0.95. However, the authors did not report on the benefits of VSP and 3DP in reducing operative time or accuracy of fracture reduction.

EPPOCRATIS offers certain advantages in the management of craniomaxillofacial trauma. First, it shortens the operative time by having the fixation plates adapted to perfected anatomy preoperatively. Plate adaptation can be taught to surgical trainees outside the operating room, saving time under anesthesia for the patient and cost. Secondly, and likely most importantly, EPPOCRATIS allows the surgeon to rehearse the surgical plan and provides the pre-bent plates as surgical guides in fracture reduction. The reduction of fractures in the actual surgery is performed with the guidance of the prebent plates rather than the traditional approach (fracture reduction followed by plate bending). Only minor plate adjustments may be necessary once anatomic reduction of fractures and occlusion is achieved. The 3DP model of the normalized anatomy is also sterilized and available as a guide during the procedure. Thus, accurate reduction of complex fractures with displacement is optimized and the incidence of post-traumatic facial deformity may be reduced. The incidence of residual facial deformity following trauma management is poorly reported in the literature [14,15]. Re-establishing pre-traumatic facial contour is challenging given the degree of scarring and bony remodeling that occurs after facial trauma treatment. The optimal timing of accurate facial trauma reconstruction is in the acute setting, not 2 years later after the above-mentioned sequalae have set in. Furthermore, facial/body dysmorphia may be developed among patients with post-traumatic facial deformity [16,17]. One of the most beneficial contributions of EPPOCRATIS to acute facial trauma treatment is ensuring the best anatomic reduction in the acute trauma setting and reducing the need for secondary surgery. An added layer of quality control may include intra-operative CT imaging, when indicated, after fracture reduction and fixation. Intra-operative CT imaging is an underutilized tool in craniomaxillofacial trauma management. Finally, EPPOCRATIS is an in-house process that can be completed in less than 48 h, allowing an expedited care of trauma patients. Bypassing outsourcing to third-party companies allows for the implementation of VSP/3DP in the acute trauma setting during the same hospitalization of trauma patients. Potential challenges with building an in-house VSP/3DP also exist. Establishing an in-house anatomic modeling and 3D printing unit requires a dedicated and code-compliant hospital space, hiring new personnel (bioengineers and technologists), training, and acquiring infrastructure (software, hardware and equipment). Furthermore, an in-house quality control process is also essential to ensure 3DP guides and models accuracy. At our institution, a strong collaboration between the plastic surgeon (B.A.S.), senior specialist radiologist (J.M.M.) and biomedical engineers allowed for experience building and more efficiency in the process. The accuracy, safety, and efficiency of EPPOCRATIS will be assessed in a future study with a matched historical cohort. As with any new technology, advances in software and product development allows for wider applicability, improved accuracy and efficiency, and reduction in costs. While the acquisition of VSP software, 3D printers, and training personnel to help in EPPOCRATIS does add costs to patient care, this technology will be less costly and more efficient in the future, which will allow wider use by facial surgeons. The history of CT imaging over the past 40 years is an example of this evolution [18]. The implementation of artificial intelligence may bypass the need for the manual segmentation of the CT scan and potentially reducing the overall time of the process and personnel costs. In addition, a more user-friendly VSP software will allow the surgeon to perform the virtual fracture reduction without the need for biomedical engineers and radiologists. Radiologists and biomedical engineers may still continue to play a role in quality control of the 3DP process, however.

## 4. Conclusions

In an era of precision surgery, the incorporation of point of care virtual surgical planning and 3D printing is beneficial in advancing facial trauma care. We describe our approach to acute craniofacial trauma utilizing EPPOCRATIS; a point of care VSP and 3DP process that relies on a multidisciplinary approach involving a plastic surgeon, radiologist, and biomedical engineer. Additional studies are needed to demonstrate accuracy, costs, and patient-reported outcomes when EPPOCRATIS vs. traditional approaches are used. Current challenges and future direction in EPPOCRATIS are discussed.

## Supplementary Materials

The following are available online at https://www.mdpi.com/article/10.3390/jcm10235640/s1, Video S1: Summary and demonstration of the different stages of EPPOCRATIS.
